# Noninvasive Ventilation and Mechanical Insufflator-Exsufflator for Acute Respiratory Failure in Children With Neuromuscular Disorders

**DOI:** 10.3389/fped.2020.593282

**Published:** 2020-10-30

**Authors:** Tai-Heng Chen, Jong-Hau Hsu

**Affiliations:** ^1^Department of Pediatrics, Kaohsiung Medical University Hospital, Kaohsiung Medical University, Kaohsiung, Taiwan; ^2^Department of Emergency, College of Medicine, Kaohsiung Medical University, Kaohsiung, Taiwan; ^3^Department of Biological Sciences, University of Southern California, Los Angeles, CA, United States; ^4^Department of Pediatrics, College of Medicine, Kaohsiung Medical University, Kaohsiung, Taiwan

**Keywords:** noninvasive ventilation, neuromuscular disorder, acute respiratory failure, mechanically assisted coughing, risk factors

## Abstract

Children with neuromuscular disorder (NMD) usually have pulmonary involvement characterized by weakened respiratory muscles, insufficient coughing, and inability to clear airway secretions. When suffering from community-acquired pneumonia, these patients are more likely to develop acute respiratory failure (ARF). Therefore, recurrent pneumonias leading to acute on chronic respiratory failure accounts for a common cause of mortality in children with NMD. For many years, noninvasive ventilation (NIV) has been regarded as a life-prolonging tool and has been used as the preferred intervention for treating chronic hypoventilation in patients with advanced NMD. However, an increasing number of studies have proposed the utility of NIV as first-line management for acute on chronic respiratory failure in NMD patients. The benefits of NIV support in acute settings include avoiding invasive mechanical ventilation, shorter intensive care unit or hospital stays, facilitation of extubation, and improved overall survival. As the difficulty in clearing respiratory secretions is considered a significant risk factor attributing to NIV failure, combined coughing assistance of mechanical insufflator-exsufflator (MI-E) with NIV has been recommended the treatment of acute neuromuscular respiratory failure. Several recent studies have demonstrated the feasibility and effectiveness of combined NIV and MI-E in treating ARF of children with NMD in acute care settings. However, to date, only one randomized controlled study has investigated the efficacy of NIV in childhood ARF, but subjects with underlying NMD were excluded. It reflects the need for more studies to elaborate evidence-based practice, especially the combined NIV and MI-E use in children with acute neuromuscular respiratory failure. In this article, we will review the feasibility, effectiveness, predictors of outcome, and perspectives of novel applications of combined NIV and MI-E in the treatment of ARF in NMD children.

## Pathophysiology Underlying Acute Respiratory Failure in Children With Neuromuscular Disorder

Neuromuscular disease (NMD) is a heterogeneous group of diseases caused by various defects from multiple sources, including skeletal muscle, motor neurons, peripheral nerves, and neuromuscular junctions (1–4). Most primary NMD is associated with an inherited gene defect and usually onset in childhood with progressive degeneration. Due to weakened either one or all of the main respiratory muscle groups and impaired coughing ability, the respiratory dysfunction represents not only a critical health issue but a frequent unmet medical need of NMD patients (2, 5, 6).

Children with NMD may have progressively developed chronic respiratory failure in the process of disease course. However, episodic attacks of acute respiratory failure (ARF) may further aggravate the already existed respiratory compromises (7). Factors posing a risk of ARF in children with NMD are usually multifactorial and occur simultaneously (8–11). Table 1 summarizes the risk levels of various NMD potentially affected by the acute respiratory compromise. According to the timing of ARF occurrence, NMD can also be classified into two main categories: (1) early-onset (may as early as in neonatal period) with rapidly progressive NMD with acute episodes of respiratory failure; (2) late-onset and slowly progressive NMD with acute exacerbations of chronic respiratory failure (12, 14).

**Table 1:** Risk levels and susceptible age groups of acute respiratory compromises in different neuromuscular disorders.

**Primarily affected age group**	**Risk level of ARF occurrence**	**Affected NMD**
At birth or within the first year of life	Usually inevitable if untreated	Spinal muscular atrophy (SMA) type 1 (if untreated)^*^ Spinal muscular atrophy with respiratory distress (SMARD) Congenital myotonic dystrophy (type 1) Infantile Pompe disease (if untreated)^*^ Some congenital myopathies (e.g., neonatal form of nemaline myopathy, minicore myopathy, and X-linked myotubular myopathy) Some congenital muscular dystrophies (CMD) (e.g., Walker-Warburg syndrome and Muscle–eye–brain disease) Some mitochondrial diseases Some congenital myasthenic syndromes
Infant-to-adult life	Very high risk	Some limb-girdle muscular dystrophy (LGMD), especially with sarcoglycanopathies (LGMD types 2C, 2D, 2E, 2F) and LGMD type 2I Some CMD, especially merosin negative types 1A, 1B, 1C Some myofibrillar myopathies (e.g., hereditary myopathy with early respiratory failure) Early-onset infantile facioscapulohumeral muscular dystrophy (FSHD) Early-onset Charcote-Marie-Tooth disease (CMTD) especially with *GDAP1* mutation Some congenital myopathies (e.g., severe recessive type of central core myopathy)
infant-to-adult life	High risk	Duchenne muscular dystrophy (DMD), usually after second decade SMA type 2 Myotonic dystrophy type 1 (DM1) Late-onset Pompe disease (LOPD) Some CMD (e.g., Ullrich type, and Fukuyama congenital muscular dystrophy) Some LGMD (e.g., calpainopathy) Some congenital myopathies (e.g., centronuclear myopathy) Bethlem myopathy Congenital myasthenic syndromes Some mitochondrial myopathies (e.g., A3243G mutation in the tRNA^Leu^ gene)
	Intermediate risk	Becker muscular dystrophy (BMD) SMA type 3 Inflammatory myopathies (e.g., polymyositis, dermatomyositis) Classical type of FSHD Some types of Charcot–Marie–Tooth disease (e.g., CMTD type 1B and 4) Some congenital myopathies Some mitochondrial myopathies Guillain–Barré syndrome (GBS) Myasthenia gravis (MG)
	Low risk	Oculopharyngeal muscular dystrophy (OPMD) Other types of CMTD Chronic inflammatory demyelinating polyneuropathy (CIDP)

* Novel therapies are currently available (e.g., enzyme replacement, antisense nucleotide, and gene therapy) to be delivered in the neonatal period.

*Data of this table are modified and summarized from references: (11–13)*.

**Figure 1:**
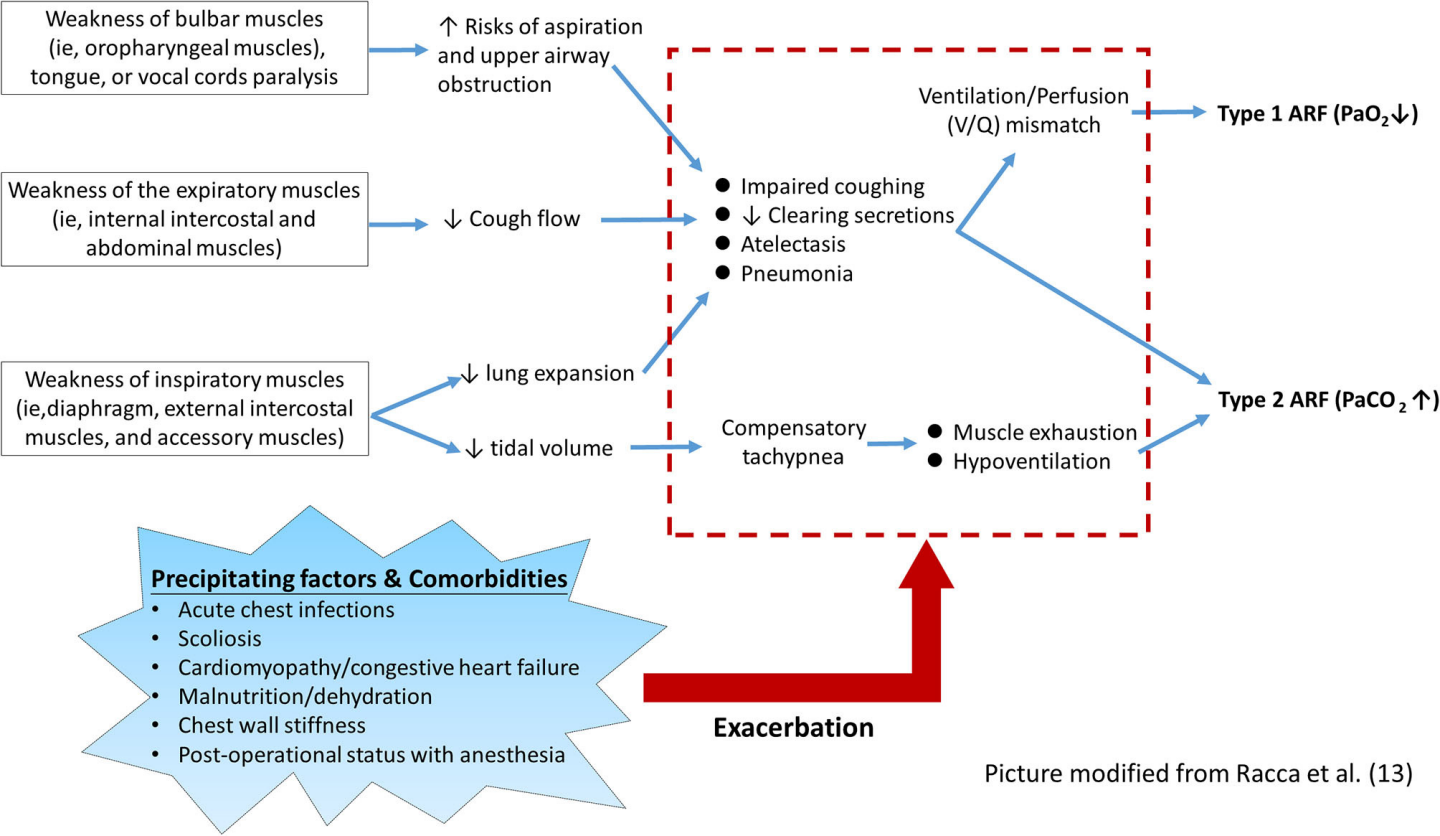
Pathophysiological mechanisms underlying ARF in children with NMD.

As shown in Figure 1, the pathophysiological mechanism of respiratory muscle groups involved in NMD patients can be summarized into three main components and several predisposing factors (6, 7, 9, 13, 14). First, the weakness of bulbar muscles impedes the protection against the risk of aspiration of the food or airway secretions, which may lead to frequent atelectasis and pneumonia (14). Additionally, weakness of bulbar muscles and tongue, and paralysis of vocal cords may cause mechanical obstruction of the upper airway, particularly in the supine position, and increase the likelihood of aspiration (9, 14). Second, weakness of the inspiratory muscles leads to reduced lung expansion and impaired coughing ability, which may lead to a ventilation/perfusion mismatch and consequent hypoxemia. Compensatory tachypnea due to small tidal volumes may further increase the mechanical load on already weakened respiratory muscles (6, 7, 13). Third, the weakness of expiratory muscles leads to ineffective coughing and encumbrance of airway secretion, which consequently increases breathing load (14).

On the other hand, other systemic involvements associated with NMD may further aggravate the impairment of lung function, which precipitate the occurrence of ARF (5, 6, 14, 15). In the advanced stage of NMD, progressive scoliosis is common and usually causes reduced chest wall compliance and unequal lung expansion. Patients with certain types of NMD, such as Duchenne muscular dystrophy and Emery-Dreifuss muscular dystrophy, frequently have cardiac involvement that may further worsen the respiratory function (e.g., pulmonary edema related to congestive heart failure) (11, 12). ARF may also occur in the perioperative period of some major surgeries, for example, correction of scoliosis or insertion of percutaneous gastrostomy. Such ARF episodes usually happen after extubation and are associated with bulbar dysfunction, postoperative pain, use of pain medications, or atelectasis caused by mucus plugging (16, 17). Malnutrition and dehydration developing during an acute illness should be aggressively intervened, as unmet caloric and metabolic needs may further aggravate ARF. Thus, each of these comorbidities necessitates multidisciplinary interventions and meticulous monitoring (5, 18–23).

In most cases, the occurrence of ARF in children with NMD is usually initiated by an upper respiratory tract infection, followed by complications of congested airway secretions, mucus plugs, and atelectasis (9, 24, 25). In addition, increased nasal airflow resistance with nasal congestion in the setting of pre-existing upper airway obstruction from bulbar dysfunction also increases respiratory muscle load in the absence of bronchial secretions. Due to community pneumonia, the decreased lung compliance and the increased workload of already weak muscles may further contribute to the onset of ARF (6). Among children with NMD, ARF is the main cause of unscheduled admissions and prolonged stay in the pediatric intensive care unit (ICU) (11, 26). Moreover, complications known to be associated with prolonged ICU stay and conventional invasive mechanical ventilator (IMV) may also contribute to high ICU mortality (27, 28). As a consequence, acute-on-chronic respiratory failure represents the most common cause of morbidity and mortality in children with NMD (9, 29).

## Noninvasive Ventilation in Childhood Acute Neuromuscular Respiratory Failure

In the past few decades, noninvasive ventilation (NIV) has been regarded as a life-prolonging tool for managing chronic respiratory failure in patients with NMD (6, 11, 21, 30). On the other hand, recent studies and guidelines have also proposed the role of NIV as a first-line intervention for ARF in NMD patients to avoid endotracheal intubation and the use of invasive mechanical ventilation (IMV) (11, 31, 32). Support for alternative use of NIV is based on concerns about the many complications of IMV use in patients with NMD. These include laryngeal edema, subglottic stenosis, barotrauma, and ventilator-associated pneumonia, leading to subsequent tracheotomy and poor quality of life (17, 33–35). Besides, long-term dependence on IMV and prolonged ICU stay are associated with nosocomial infections, aspiration, atelectasis, thromboembolic events, contractures, and bedsores, all of which can lead to high mortality in NMD patients (8). In this regard, emerging evidence supports the alternative NIV administration to manage ARF in patients with NMD (36–38). Indeed, several studies have indicated several potential benefits of NIV in treating ARF of NMD patients, including shortening the ICU and hospital stay, facilitating extubation, and improving the overall survival (16, 39–42).

## Role of Aggressive Secretion Management in Managing ARF of NMD Children

Mucociliary clearance is generally not affected by NMD, except for damage to the ciliary epithelium due to repeat aspiration or acute chest infection (43). Aggressive secretion clearance is crucial for children with NMD to avoid progression to severe respiratory compromises during respiratory infections (44, 45). Also, excessive secretion has been regarded as a major risk factor causing NIV failure in treating ARF of NMD patients (6, 46, 47). Therefore, facilitating secretion clearance and normalizing gas exchange by augmenting cough ability is the mainstay to treat ARF in children with NMD (48).

**Figure 2:**
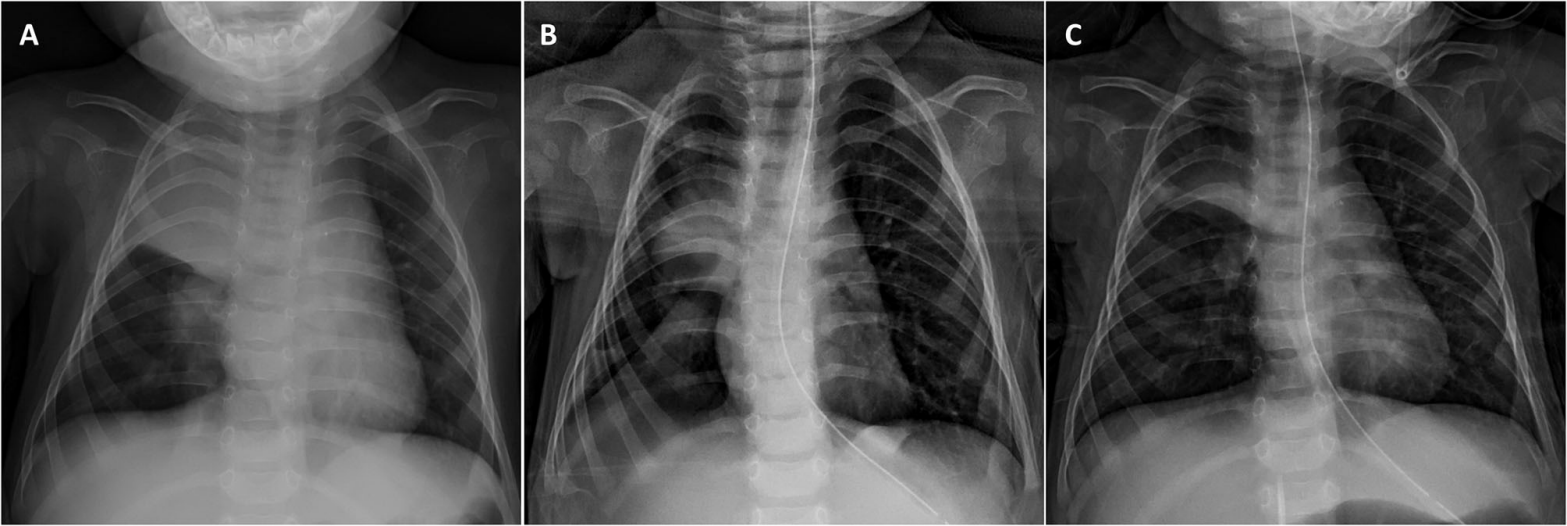
Resolution of right upper lobe opacification in an infant with severe type 1 spinal muscular atrophy (SMA) after combining NIV and MI-E. **(A)** Chest X-ray on admission showing right lung pneumonia with significant atelectasis complicated by copious secretions. **(B)** A significant improvement was found after 2-days treatment, with a resolution of atelectasis. **(C)** A progressive improvement of the pneumonic patch was observed on day 7 when discharged from PICU.

Although NMD patients rarely achieve sufficient chest and abdomen pressure due to the weakness of the intercostal and abdominal muscles, the coughing can be augmented manually or mechanically. Among various coughing-assist techniques, the mechanical insufflator-exsufflator (MI-E) represents the most powerful tool that can promote the most effective peak flow to expel mucus plugging and resolve atelectasis (7, 49, 50). MI-E can deliver a brief positive inspiratory pressure through a mask, mouthpiece, tracheostomy, or endotracheal tube to fully expand the chest, allowing air to enter the distal end of the mucus plugging, and then applying negative pressure, resulting in expiratory “cough” flow to remove airway secretions (45). A previous study showed that MI-E is superior to manual cough assistance in increasing cough flow in healthy subjects as well as in patients with amyotrophic lateral sclerosis (ALS), regardless of bulbar weakness (51). The additional use of MI-E helps to resolve excessive secretions and eliminate the risk of NIV failure in treating ARF of NMD patients. Therefore, recent evidence suggests that combining NIV and MI-E can be used as the first-line treatment for ARF in children with NMD (14, 44, 45, 47, 50). Our experiences also show that it can effectively treat ARF even for the most severe types of NMD (Figure 2).

## Effectiveness of NIV in Post-Extubation Support for Children With NMD

**Figure 3:**
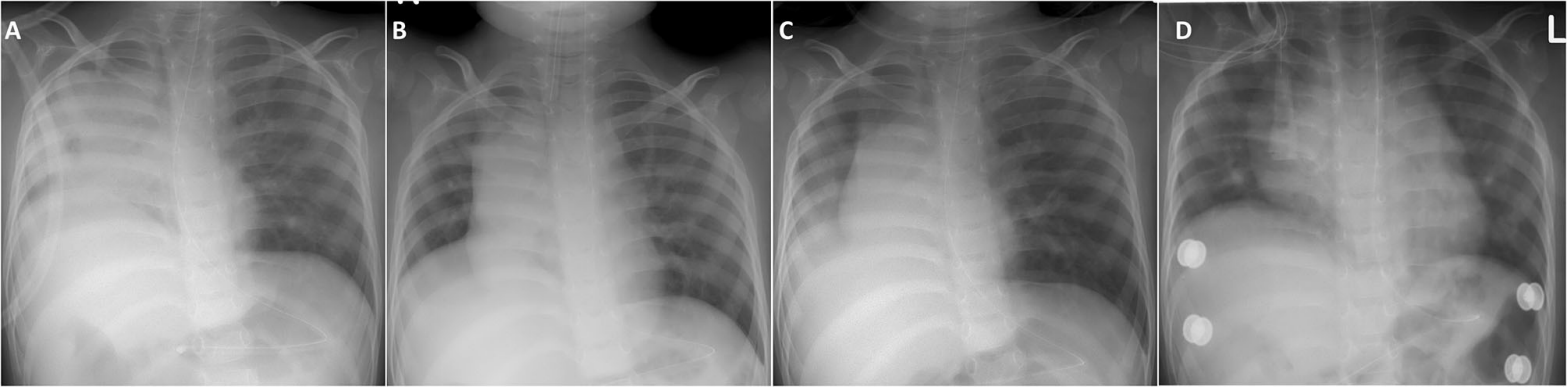
Demonstration of chest X-ray in a toddler with congenital myopathy who immediately received NIV and MI-E for post-extubation respiratory support. **(A)** Previously failed extubation in another hospital was related to frequent right lung atelectasis and mucus plugging developing soon after extubation. **(B)** In our hospital, appropriate expansion of both lungs were noted before extubation. **(C)** Day 2 post-extubation showed mild right lung infiltration without atelectasis. **(D)** Discharge from PICU on day 7 post-extubation showed re-expansion of both lungs.

After recovering from an acute illness or surgery requiring sedation, a considerable number of NMD patients may not pass the IMV-dependent weaning tests, resulting in a high failure rate of extubation (26–28). Post-extubation ARF in NMD patients shares several pathomechanism features with episodic ARF, such as weak respiration drive, airway mucus-plugging due to difficulty in expectorating secretion, mostly categorized as type 2 (hypercapnic) ARF (28). The advent of active NIV support reduces the need for extensive weaning trials before extubation, which requires prolonged pressure support and spontaneous breathing. Some studies have validated that prompt NIV and MI-E use after extubation can significantly eliminate the risk of reintubation in NMD patients (40, 52, 53). There is a general agreement that, if not contraindicated (e.g., uncontrolled airway secretions or severe bulbar dysfunction), patients with chronic NMD should be extubated directly to NIV combined with MI-E (28, 54). The effectiveness of this NIV support is significant in preventing reintubation in young children with NMD (Figure 3).

## Review of Clinical Studies on NIV for the Treatment of Acute-On-Chronic Neuromuscular Respiratory Failure

There are relatively few prospective studies on the management of NMD patients with ARF, which may be because most chronic NMDs are rare diseases, making it difficult to recruit patients. As shown in Table 2, evidence that NIV can help avoid intubation of patients with chronic NMD during the ARF episodes comes from 11 non-randomized observational studies of a total of 178 subjects (age range 2 months to 69 years), of which most subjects are known to be under 25 years (36, 37, 39–42, 52, 53, 56, 57). However, in most studies, there are few descriptions of methods to manage airway secretions, and its role in contributing to the success of NIV in treating ARF is not well defined (45). Even though an increasing number of studies have recognized the benefit of combined NIV and MI-E use in the ARF management and facilitation of extubation in adult NMD patients (37, 40, 54), similar studies on the pediatric NMD populations are scarce. In heterogeneous pediatric populations, several risk factors for predicting the failure of NIV treatment fo ARF have been reported (11, 32, 44), but it is still unclear whether similar factors exist in a specific pediatric NMD population.

**Table 2:** Noninvasive airway approaches for patients with NMD with acute on chronic respiratory failure.

**References**	**Study Design**	**Number of NMD patients (age)**	**NMD diagnosis (n)**	ARF types^*^ (n, %)	**NIV/interface/ secretion clearance**	**Success rate and main findings**	**Predictor of NIV failure**	**NIV Complications (n)**	**Limit**
Padman et al. (39)	Monocenter retrospective study	11 patients; (range: 4-21 y)	DMD (7), SMA (2), SCI (1), nonspecific myopathy (l)	Type 2 (11, 100%)	BLPAP via nasal mask	• NIV success rate (no intubation): 91 % • Improved RR, PaCO_2_, serum bicarbonate, and length of hospitalization after NIV use	None identified	No major complications	Hypoxic ARF and significant difficulty handling secretions
Birnkrant et al. (55)	Monocenter retrospective study	8 patients (range 1-18 y)	DMD(5), SMA(3)	Undefined ARF, including 3 post-extubation ARF	BLPAP via nasal interface	• Allowed weaning from an invasive airway: 100% effective in avoiding ETI or facilitating extubation	None identified	NA	Non described
Niranjan and Bach (40)	Monocenter retrospective study	10 patients (median: 17 y; range: 13-21 y) vs. 7 historical controls	DMD (8), SMA (1), SCI (1)	Type 2 (10, 100%), including 6 post-extubation ARF	BLPAP via mouthpiece or nasal interface + MI-E	• NIV success rate (no intubation): 100% • Shorter hospital stay in NIV group than historical control	None identified	NA	Non described
Bach et al. (56)	Monocenter retrospective study	11 children with 28 ARF episodes (median: 6 m; range: 2-11 m)	SMA type 1 (11)	Post-extubation ARF (28, 100%)	BLPAP via nasal interface+ MI-E for post-extubation support	• NIV success rate (no intubation): 82 % • NIV can facilitate extubation for type 1 SMA children even with severe bulbar muscle weakness	None identified	NA	Non described
Vianello et al. (36)	Monocenter prospective case-control study	14 patients (median: 24 y; range: 10-69 y) vs. 14 historical controls	DMD (7), ALS (4), CMD(1), HMSN (1), CM(1)	Type 2 (14, 100%)	E = BLPAP via nasal interface + cricothyroid-mini-tracheostomy; C = IMV via ETI	• NIV success rate (no intubation): 71% (14% mortality rate) vs. 21% of controls (57% mortality rate) • Lower mortality and complications, and shorter ICU stay of NIV group than controls • NIV combined with cricothyroid-mini-tracheostomy for secretion clearance was well tolerated without significant complications	None identified	No major complications	Severe bulbar involvement
Vianello et al. (37)	Monocenter prospective case-control study	11 patients (median: 31 y; range: 16-64 y) vs. 16 historical controls	DMD (4), SMA (3), ALS (2), LGMD(1), FSHD (1)	Type 2 (11, 100%)	E = BLPAP via nasal interface+ MI-E+CPT; C = BLPAP+CPT	• NIV success rate (no intubation): 82 vs. 37% of controls • No serious side effects and well-tolerated in all subjects with MI-E use	None identified	Gastric distension (1), epistaxis (1)	
Servera et al. (41)	Monocenter prospective cohort study	17 patients (48.7 ± 20.9 y)	ALS (11), DMD (4), transverse myelitis (1), nonspecific myopathy (1)	Type 2 ARF (17, 100%)	BLPAP via nasal/oronasal interfaces + MI-E	• NIV success rate (no intubation): 79.2% • Severe bulbar involvement limited NIV effectiveness	Bulbar dysfunction	NA	Severe bulbar involvement NIV/MI-E performed in non-ICU settings
Piastra et al. (42)	Monocenter prospective observational cohort study	10 children (4.1 ± 4.5 y; range 3 m-12 y)	SMA type 1(2), CMD –Ullrich (1), CM-nemaline CM (1), MG (2), mitochondrial myopathy (1), spinal cord hamartomatosis (1), nonspecific myopathies (2)	Type 2 (5, 50%); Type 1 (2, 20%); mixed/undefined (3, 30%)	BLPAP via facial mask or helmet+ CPT	• NIV success rate (no intubation): 80% • Hypercarbic ARF resolved within 6 h of NIV use • Oxygenation markers improved rapidly after NIV introduction	Airway obstruction	No major complications	Copious tracheal secretion needing frequent suction
Dohna-Schwake et al. (53)	Monocenter retrospective study	15 children (median: 6 y)	SMA (6), DMD (3), Pompe disease (2); CMD (2), myopathy (1), myotonic dystrophy (1)	Undefined ARF, including 2 post-extubation ARF	CPAP via mask	• NIV success rate (no intubation): 87% • Improved HR, RR, blood pH, PaCO_2_, and SaO_2_ after 1-2 h of NIV use in the success group	Low pH at 1–2 h after NIV	midface skin ulcers and gastric distension	3 patients requested “do-not-intubate-status”
Chen et al. (57)	Monocenter prospective observational cohort study	15 children with 16 ARF episodes (mean: 8.1 y; range 3 m- 18 y)	SMA (6), DMD (2), CM (2), MM (2), HMSN (2), LGMD 2I (1)	Type 2 (15, 94%) including 1 post-extubation ARF; Type 1 (1, 6%)	BLPAP via nasal/oronasal or facial mask + MI-E	• NIV success rate (no intubation): 75% • Improved blood pH, and PaCO_2_ after 12 h of NIV use in the success group	Fewer decrement of RR after 3 h of NIV use	No major complications	
Chen et al. (52)	Monocenter prospective observational cohort study	56 NMD patients (44 children) with 62 ARF episodes; median: 13 y; range: 2 m-39 y)	SMA (32), DMD (14), CM (6), CMD (4), MM (4), HMSN (1), SMARD (1)	Type 2 ARF (53, 85%) including 23 post-extubation failure; Type 1 ARF (9, 15%)	BLPAP via nasal/oronasal or facial mask + MI-E	• NIV success rate (no intubation): 86% • Improved HR, RR, blood pH, and PaCO_2_ after 4 h of NIV use in the success group • Shorter PICU and hospital stay of success group	RR decreased at 4 h; pH increased, and PaCO_2_ decreased at 4-8 h after NIV	No major complications	Initial checking blood gases at a later point of 4–8 h after NIV

* Type 1 ARF, Hypoxemic ARF; Type 2 ARF, hypercapnic ARF.

NMD, neuromuscular disorders; NIV, non-invasive ventilation; ARF, acute respiratory failure; BLPAP, bi-level positive airway pressure; MI-E, Mechanical insufflator-exsufflator; E, experiment; C, control; CPT, chest physical treatments; DMD, Duchenne muscular dystrophy; SMA, spinal muscular atrophy; SCI, spinal cord injury, HMSN, hereditary motor and sensory neuropathy; CMD, congenital Muscular Dystrophy; CM, congenital Myopathy; MG, myasthenia gravis; MM, mitochondrial myopathy; SMARD, spinal muscular atrophy with respiratory distress; LGMD 2I, limb-girdle muscular dystrophy type 2I; NA, Not available.

*CPAP, Continuous positive airway pressure*.

However, only one randomized controlled study has investigated the efficacy of NIV in treating children with ARF but has excluded children with underlying NMD (31). Two recent studies reported by the same team described the protocol and effectiveness of a combination of NIV with MI-E in treating ARF of children with chronic NMD (52, 57). The pilot study of children encompassing various NMDs has demonstrated the feasibility of this combined noninvasive approach. The following research on a larger cohort of NMD patients further verified its safety and effectiveness. Overall, combining the data of these two studies on 71 NMD patients shows that timely implementation of NIV and MI-E can avoid intubation or reintubation in 75–86% of ARF events, of which 80% are pediatric cases. The PICU and hospital stay of children successfully rescued through NIV/MI-E is shorter than that of children who received intubation. Besides, several predictors of NIV failure were identified, including physical parameters (changes in respiratory rate) and laboratory variables (changes in PaCO_2_ and pH value of arterial blood gas).

## Combined NIV and MI-E in ARF Treatment of NMD Children

The interface connects the ventilator tubing to the patient to deliver pressurized gas to the airway during NIV administration. It may take several attempts to find a suitable interface, but this is the key to successfully treating ARF in NMD children with NIV while minimizing air leakage, maximizing patient comfort, and synchronizing with the ventilator (44, 58, 59). However, although interface tolerance is a pivotal factor associated with NIV success, comparative data on the interface of infants and young children is scarce (60).

A transparent interface is highly recommended to ensure correct positioning and enhance patient monitoring (59). The medical team should be well trained to select the most suitable interface individualized for each critically ill child (61). As proof of principle, the smallest interface with the least air leakage should be selected to minimize the dead space. For infants, nasal interface (nasal cannula, nasal prong, or nasal mask) is recommended the interface of first choice (6, 44). Otherwise, choosing the right interface for older children is usually based on available materials and training of an experienced medical team, not on scientific data. Generally, in older children and young adults with ARF, full oronasal face masks are preferable to nasal interfaces because of better tolerance and a better sealing with less air leak (60, 62). Although some studies have shown that the feasibility and effectiveness of helmets in infants and young children, the experience of using helmets as interfaces in children is even rarer (62). It should be kept in mind that there is no single interface suitable for all situations, and the use of these interfaces in NMD children, especially in the critical care setting, requires better evidence support (59).

Recommendations for the initial setting of NIV are mainly based on clinical experience and expert consensus as there are no consistent data on optimal settings. If not contraindicated as the list aforementioned, the initial settings chosen should be disease and device-specific. Importantly, the information regarding the potential contraindications or complications related to NIV administration in the NMD patient population should be addressed (6, 11). Generally, the administration of NIV support should be set low initially to allow patient acclimation and then increase according to the physiologic needs and patient tolerance. According to our protocol specialized for NMD children, bilevel positive airway pressure (BLPAP) with an adequate interface is always effective in rescuing ARF (52, 57). Especially during an acute chest infection, NIV should be used more intensively for these patients. Under adequate approaches of secretion clearance, supplemental oxygen may be added to NIV to maintain appropriate oxygenation. However, if the patient becomes almost whole-day dependent on NIV during an acute event, consider alternating masks to prevent pressure sores and alternate day and night between two ventilators of the same model so as not to run a ventilator continuously for days. The MI-E can be applied either in combination with NIV through a full-face mask or solitarily used in intubated patients via the endotracheal or tracheostomy tube with the cuff inflated. If applicable, supplementary manual augmentation of cough may be applied intermittently, followed by MI-E use.

In addition to noninvasive airway approaches, all other sensible standard measures can be taken during ARF episodes. These approaches include adapting a low threshold to deliver broad-spectrum antibiotics, adequate hydration, and attention to nutritional support. Humidification of the ventilator is often beneficial in reducing sputum viscosity and mobilizing secretions. Therapies of nebulized bronchodilator or systemic steroid may be considered if evidence of asthma or asthmatic bronchitis (10, 60).

From the perspective of chronic respiratory care, proactive use of NIV, and cough assistant MI-E in NMD children has been shown to reduce the rate of hospitalization and ICU admission (63–65). The familiarity of NMD patients with NIV use can help the effectiveness of NIV in the treatment of ARF (60). Several studies have shown that prior training of NIV and MI-E at home can contribute to a higher success rate in acute care settings (14, 52, 66). In this regard, the proactive use of NIV and MI-E in the routine respiratory care of children with chronic NMD may also be beneficial (65).

Besides MI-E, high-frequency chest wall oscillation (HFCWO) has recently been proposed as a potential intervention used to facilitate secretion clearance in NMD patients. HFCWO delivers pressure to the chest wall accompanied by high-frequency vibration, which shows to move secretions from peripheral airways toward more central airways (67). However, the safety and effectiveness of HFCWO have not been well studied in managing ARF of NMD children, and its benefit in acute care settings is unclear (68). There is still a lack of data on the safety and effectiveness of NMD infants and young children known to be more susceptible to consistent and high frequent oscillation waves. Further research on HFCWO in NMD children is needed.

## Contraindications and Complications of NIV and MI-E

The patient selection remains the most critical factor for the success of NIV in treating ARF. The contraindications to the NIV use include hemodynamic instability, severely decreased consciousness level, severe bulbar dysfunction (i.e., absence of gag reflex, or vocal cord paralysis), un-drained pneumothorax, facial deformity or injuries, recent surgery of facial, upper airway, or upper gastrointestinal tract, intolerance to NIV interface, multi-organ failure, life-threatening hypoxemia (PaO_2_ <60 mmHg with FiO_2_ > 0.6), and lack of familiarity of health-care provider with NIV operation (6, 14, 62, 69, 70).

In general, NIV is a safe approach in managing ARF of infants and children with NMD, and the adverse effects described are minor (71). However, similar to any ventilation therapy, there are some adverse reactions and severe complications worthy of understanding. Reducing complications of NIV and MI-E largely depends on the well-trained and experienced staff of a multidisciplinary care team (21, 44, 62, 72). Gastric distension may occasionally occur, which can be ameliorated by nasogastric tube insertion and keeping adequate enteral feeding. Barotrauma may occur, but the risk is extremely low during NIV and much lower than during mechanical ventilation (73). For patients with hypovolemia, NIV should be used with caution, because NIV can cause an additional increase in intrathoracic pressure, which may result in a decrease in venous return (preload) and further deteriorate cardiac output (74).

Agitation may develop, especially during the initial interface placement on a child, but it is not necessary to discontinue NIV for this reason. Pharmacological sedation may be required, especially for children with NMD who receive NIV for the first time (75, 76). Choosing a more comfortable interface and fine-tuning NIV settings can reduce the need for sedatives (62). Other related complications include skin lesions, discomfort, claustrophobia, nasal mucosa trauma, and conjunctivitis, which may be prevented by a sophisticated selection of appropriate interface, alternating interface intermittently, and humidification of the ventilator (59, 60).

## Conclusions

The care of chronically progressive NMD has evolved significantly in the last decade, and many NMD children are now achieving prolonged survival through the advances in novel treatments (e.g., gene and molecular therapies) as well as respiratory care. However, there is still no consensus on the timing and limitations of NIV use in the treatment of ARF in children with NMD. Therefore, the administration protocol must be integrated with individualized clinical judgment. NMD usually includes various diseases of different severity, and the pathomechanism of ARF may vary with the type of NMD. Thus, it is unclear whether certain types of NMD may be more sensitive to NIV treatment for ARF. The variety and complexity of specific problems presented by different NMD necessitate separate remarks on the early recognition and adequate management of ARF in children with NMD. More future researches designed specifically for the pediatric NMD population are still needed, and several issues remain to be clarified.

## Author Contributions

T-HC and J-HH contributed to conception and design, acquisition of data, revising the manuscript critically for relevant intellectual content, and final approval of the version to be published. All authors read and approved the final manuscript.

## Conflict of Interest

The authors declare that the research was conducted in the absence of any commercial or financial relationships that could be construed as a potential conflict of interest.
